# Nutritional assessment of community-dwelling older adults in rural Nepal

**DOI:** 10.1371/journal.pone.0172052

**Published:** 2017-02-14

**Authors:** Saruna Ghimire, Binaya Kumar Baral, Karen Callahan

**Affiliations:** 1 Department of Environmental and Occupational Health, School of Community Health Sciences, University of Nevada, Las Vegas, Nevada, United States of America; 2 Department of Biochemistry, Nepal Medical College and Teaching Hospital, Kathmandu, Nepal; Oslo Universitetssykehus, NORWAY

## Abstract

**Background:**

Demographic transition in Nepal, like in many developing countries, has resulted in a burgeoning elderly population whose health status is not currently monitored. One pillar of health is adequate nutrition. Yet, little is known about the nutritional health status of the elderly in Nepal. The financial, material, and personnel limitations in Nepal’s health delivery services necessitate health screening instruments that require minimal clinical staff and resources. To our knowledge, no such nutritional assessment tool has been validated in Nepal. Therefore, our aims are two-fold: To assess the nutritional status of the elderly population in one typical Nepali village, Okharpauwa, in Nuwakot District, Nepal; and concurrently, to validate the Mini Nutritional Assessment (MNA) tool.

**Methods:**

A cross-sectional field study was conducted with a sample of 242 elderly people in Okharpauwa, Nepal to obtain prevalence of malnutrition. Differences in demographic and lifestyle factors between these who were malnourished, those at risk of malnourishment, and those who had adequate nutritional status were analyzed. The MNA tool was evaluated using receiver operating characteristic (ROC) curve analysis; sensitivity, specificity, and diagnostic accuracy were calculated.

**Results:**

111 males and 131 females, with a mean age of 69.8±7.4 years, participated in this study. The mean BMI of the participants was 21.4±3.9 kg/m^2^; the mean MNA score was 19.3±4.2. BMI was significantly correlated with the total MNA score (r = 0.58; p<0.001). The diagnostic accuracy, sensitivity and specificity of MNA were 81%, 86% and 67% respectively. Of the 242 elderly sampled, 24% were malnourished and 65% were at risk of malnutrition. Malnutrition was more prevalent among females (29%) than males (18%), and most prevalent among the marginalized Dalit ethnic group (40%). Elderly persons who were married and literate had better nutritional health than their counterparts.

**Conclusions:**

The MNA appears to be a valid and sensitive tool for rapid nutritional screening of the elderly in Nepal. The prevalence of malnutrition was high among Nepalese elderly in the Okharpauwa VDC, which requires urgent health monitoring and management attention.

## Introduction

By 2050, the global population of elderly people is projected to reach 2 billion, a dramatic threefold increase from 739 million in 2009 [1]. Moreover, 79% of the world’s older people will live in less developed regions [1]. According to the Nepali census, the population of elderly, defined by the Nepali Senior Citizens Act [2] as persons 60 years of age and above, grew from 1.5 million in 2001 [3] to 2.2 million in 2011 [4]. This represents a 3.5% growth rate for the elderly, surpassing the 2% overall population growth rate [5]. Therefore, Nepal needs to prepare for the obligation to address the specific social, psychological, economic, and particularly health needs of this aging population.

Good nutrition is the foundation for a healthy life. Malnutrition is associated with higher rates of morbidity and mortality [6], as well as increased health care expenditures [7–9], and prolonged hospital stays [10, 11]. According to World Health Organization estimates, 1 in 6 persons globally was affected by malnutrition in 2015 [12]. Geriatric populations are uniquely susceptible to malnutrition due to the association of aging with factors that influence nutritional status: decreased appetite, decreased energy expenditure, weight loss, taste and smell changes, feelings of loneliness and depression, difficulty chewing, fatigue and co-existing morbidities [13]. For elderly people, the potential consequences of malnutrition include a decline in functional status, impaired muscle function, decreased bone mass, immune dysfunction, anemia, reduced cognitive function, increased susceptibility to infection, and poor wound healing [14]. In Nepal, poverty and inadequate government social security benefits, combined with the lack of knowledge about nutrition, further contribute to the vulnerability of the elderly to malnutrition.

To date, information regarding the nutritional status of the elderly in Nepal is scarce. The ongoing Demographic and Health Survey [15–17], as well as Nepal’s National Nutritional Plan and Strategy [18, 19], while covering the nutritional health of women and children, do not include this parameter for elderly citizens. In 2010, the Nepal Geriatric Centre [5] published a status report highlighting the lack of knowledge about the health, nutritional status, and overall quality of life of elderly people in Nepal, recommending research in these areas in order to effectively plan programs and interventions that will maximize the quality of life of Nepal’s geriatric population. To address one of these gaps in knowledge, this study aims to estimate the prevalence of malnutrition among the elderly in Nepal.

Thorough and comprehensive clinical assessments of nutritional status, while undoubtedly ideal, are time-consuming, expensive, and often inconvenient for the elderly. Therefore, brief nutritional screening tools that provide an effective and inexpensive way to detect malnutrition are preferred. To our knowledge, no such screening tool has been validated in Nepal. One widely used tool is the Mini Nutritional Assessment (MNA), which has many advantages for use in Nepal [20, 21]. First, the MNA can be easily administered by peripheral or primary level health staff in rural areas, without the need for biochemical testing or specific training in nutrition [20, 22]. In Nepal, primary level health personnel, who have little or no clinical background, bear most of the burden of providing health services. Additionally, limited availability of laboratory services in Nepal makes comprehensive clinical nutritional assessments unfeasible, especially in rural settings. Secondly, the MNA has already been widely used, as well as validated, across diverse settings, including other South Asian countries [23–26]. However, to the best of our knowledge, the MNA has not been validated in Nepal. Therefore, this study has two aims: (1) to validate a Nepalese version of the MNA tool; (2) to use the MNA to estimate the prevalence of malnutrition among elderly people in the Okharpauwa Village Development Committee (VDC) in the Nuwakot District of Nepal.

## Methods

The reporting of our study followed the Strengthening the Reporting of Observational Studies in Epidemiology, STROBE, guidelines [27].

### Study setting

This study was conducted in January-March, 2016 in Okharpauwa Village Development Committee (VDC), a remote hilly village in Nuwakot District, located 18 kilometers northwest of Nepal’s capital city, Kathmandu. Okharpauwa is one of Nepal’s 3,157 rural VDCs, selected conveniently due to its relative proximity to Kathmandu. Moreover, the majority (85%) of the geriatric population in Nepal resides in a rural area [5]. This village has a total population of approximately 8000 people living in 1500 homes [4]. According to the 2011 census, the total number of people ages 60 years and above in Okharpauwa was 622, of which 338 were male and 284 were female [4].

### Ethics and consent

This study, including all aspects of consent procedures and data collection protocol, received Ethical Approval from the Institutional Review Committee (IRC) at Nepal Medical College Teaching Hospital (NMCTH), in Kathmandu, Nepal. A detailed verbal explanation of the purpose of the study as well as the procedures to be followed, including collection of anthropometric measurements, was given to the respondent; subsequently, informed verbal consent was obtained by the surveyor and recorded on the survey instrument. Verbal rather than written consent is common in Nepal [15, 16, 28]; for example, the Protocol for the Nepal National Micronutrient Survey takes verbal consent due to low levels of literacy and the association of signatures/thumbprints with strictly legal documents [28]. The data were collected after verbal consent was documented. All respondents participated voluntarily. The identity of respondents was kept confidential.

### Study procedure

Given the finite population of a total of 622 elderly persons residing in Okharpauwa [4], the sample size of 242 individuals was calculated using StatCalc in Epi Info 7 [29] with 5% alpha, or Type I error, 5% margin of error and 50% prevalence.

A cross-sectional house-to-house survey was conducted among the community-dwelling elderly, selecting households by systematic random sampling. Surveyors, supervised by the second author, were first-year medical students who were provided with a one-day extensive orientation on the MNA tool, sampling strategy, and data collection techniques. Surveyors started from the center of the village, stopping to collect data from every other house. There were no refusals. If two or more eligible participants were in one household, as is common in Nepal, only one was selected by simple random sampling lottery method. If an eligible participant was not present in the selected house, then data was sought first from an eligible participant in the adjacent house to the right, then to the left if there was not an eligible participant in the house to the right. Criteria for eligibility included being at least 60 years old and a permanent resident of Okharpauwa, defined as at least one year of residence. Participants who were too frail physically and/or mentally to respond were excluded. Surveyors stopped when the sample size of 242 elderly persons had been achieved.

### Data collection and variables

Individual interviews using the MNA were conducted at each participant’s home by the surveyors. As is protocol for translations [30], the MNA tool was first translated from English to Nepali by the second author, and then verified by back-translation to English by an experienced translator. One question was changed slightly in order to assess independent living in a more culturally appropriate manner, as very few elderly in Nepal live in nursing homes [5]. Therefore, instead of “Lives independently (not in nursing home or hospital)”, as stated in the original MNA, we asked, “Do you need help from others to perform your routine daily activities?” The MNA consists of 18 items across four assessment dimensions: anthropometric, global, dietary, and subjective [31]. Cumulative scores range from 0 to 30. For the assessment of nutritional status, categorized into three types, an MNA score of less than 17 indicates malnutrition, scores between 17 and 23.5 indicate the subject is at risk of malnutrition, and any score of 24 or higher is considered normal nutritional status [31].

Four anthropometric assessments, all shown to be valid indicators of nutrition status among the elderly in developing countries [26, 32–34], were taken by the surveyor while participants wore light clothing and were barefoot: height, weight, calf circumference (CC), and mid-arm circumference (MAC). Height was measured using a mechanical stadiometer; weight was measured with a digital weighing scale. BMI was calculated as weight in kg / (height in m)^2^. Calf circumference was measured on seated participants with an inextensible tape at several locations to find the maximal bare calf circumference. For mid-arm circumference, the participant’s forearm was held in horizontal position to locate and mark the mid-distance between the acromial surface of the scapula and the olecranon process of the elbow. With the arm hanging freely at the side, circumference at that marked arm mid-point was measured. Per instructions in the MNA guide [20], BMI, CC, MAC and MNA were converted from a continuous scale to categories.

Data on existing comorbidities, drug prescriptions, loss in weight and/or appetite in past three months, mobility, neuropsychological problems, food and fluid intake, and self-perceived nutritional and health status were self-reported. Additional sociodemographic and lifestyle variables including age, sex, ethnicity, religion, educational status, marital status, smoking status and alcohol consumption were collected, all by self-report. For ethnicity, the Nepal Health Management Information System’s classification was used; related categories were combined to obtain three ethnic groups: Upper Caste, Janjatis and Dalit. Based on historical association with the caste system [35], persistent in driving disparities between ethnic groups [35], these three ethnic groups would be expected to generally represent higher, medium, and lower social status, with the Dalit representing the most marginalized of all groups [35, 36]. Educational status was categorized into three groups: illiterate, informal, and literate. Informal education referred to having some literacy skills but no formal education. Smoking habit was categorized into three mutually exclusive groups: never smoker, current smoker, (smoking for at least a year), and former smoker, (quit over one year ago). Alcohol drinking patterns were defined as never, infrequent, and frequent; frequent was defined in this local context as having at least one drink weekly.

### Data processing and statistical analysis

Data were managed in EpiData [37]. To ensure the accuracy and quality of data entry, 10% of total data were randomly selected and rechecked manually by a second person. This process was repeated until no errors were found. Statistical analyses were performed in IBM SPSS22 (Statistical Package for Social Sciences) for Windows (SPSS Inc. Chicago IL, USA).

For the MNA tool validation, Spearman’s rank correlation between total MNA score and each of the 18 items included in the MNA were calculated. In receiver operating characteristics (ROC) curves analysis, validity of the MNA was evaluated against BMI as the reference. BMI has been found to be a useful tool in clinical and public health practice for assessing the nutritional status of adults [38] as well as the elderly [33, 39, 40]. Additionally, BMI is widely used in nutritional surveys [41, 42], and is a proxy indicator of socioeconomic status, particularly in developing countries [43, 44]. In ROC curves analysis, diagnostic accuracy, sensitivity and specificity of the MNA were evaluated, using MedCalc for Windows, version 15.11 (MedCalc Software, Ostend, Belgium). Diagnostic accuracy of the MNA, denoted by area under an ROC curve (AUC) and ranging from 0–1, was defined as follows: Acceptable, 0.7–0.8; Excellent, 0.8–0.9; and Outstanding, >0.9 [45]. Youden index J criteria [45, 46] were used to define sensitivity and specificity cutoff points.

To assess nutritional status, prevalence of the three nutritional categories—malnourished, at risk of malnourishment, and normal nutritional status—were calculated. The Shapiro-Wilks test was used to test the normality of variable distributions. Comparisons of means between the nutritional status groups were done by ANOVA, and frequency distributions were evaluated by Pearson’s chi square (χ^2^) test or the Fisher-Freeman-Halton Exact test, as indicated by variable distribution. For all statistical tests, two-tailed p-values <0.05 were considered statistically significant.

## Results

Two hundred forty-two senior citizens, 131 females and 111 males, participated in the study. The overall response rate for each MNA question was 100%. The mean age of the participants was 69.8±7.4 years of age. A majority of the participants were Hindu (75%), married (68%), illiterate (72%), and Upper caste (57%). At the time of the study, 44% of the participants had never smoked and 69% did not consume alcohol. The mean height, weight and BMI of the participants were 1.5±0.1m, 48.1 ± 9.1 kg and 21.4 ± 3.9 kg/m^2^, respectively. Descriptive characteristics of the participants are provided in Table 1.

**Table 1:** Characteristics of the 242 elderly participants.

Characteristics	Participants
	**mean ± SD**
**BMI**	21.4 ± 3.9
**Age**	69.8±7.4
	**n (%)**
**Gender**	
Male	111 (45.9)
Female	131 (54.1)
**Ethnicity**	
Upper caste	138 (57.0)
Janajati	77 (31.8)
Dalit	27 (11.2)
**Religion**	
Hindu	181 (74.8)
Buddhist	58 (24.0)
Christian	3 (1.2)
**Education**	
Literate	53 (21.9)
Informal	14 (5.8)
Illiterate	175 (72.3)
**Marital status**	
Married	165 (68.2)
Separated	11 (4.5)
Widowed	66 (27.3)
**Smoker**	
Never	105 (43.6)
Current	93 (38.6)
Former	43 (17.8)
**Drinker**	
Never	168 (69.4)
Infrequent	62 (25.6)
Frequent	12 (5.0)

Abbreviations: SD, standard deviation; BMI, body mass index.

### MNA tool validation

**Fig 1:**
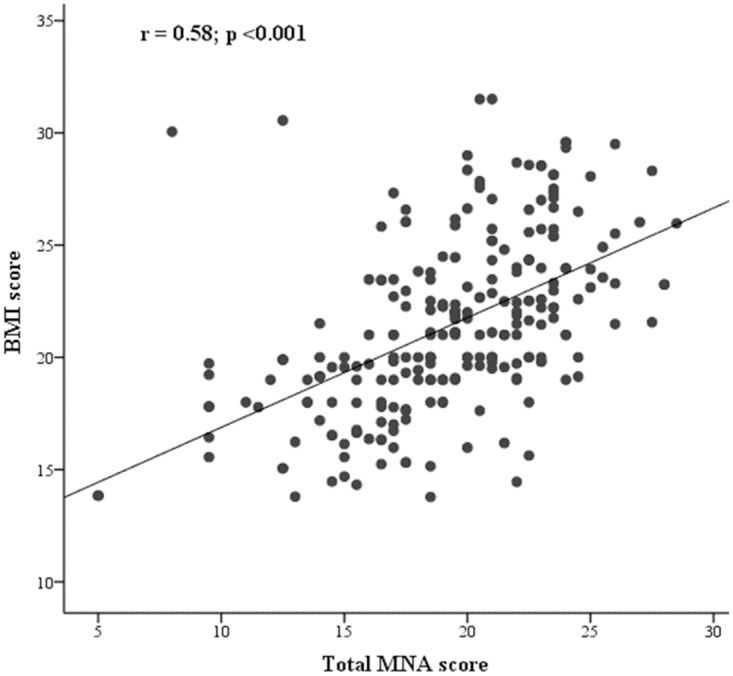
Scatter plot and Spearman correlation coefficient (r) of BMI and total MNA score. BMI, body mass index; MNA, mini nutritional assessment.

**Fig 2:**
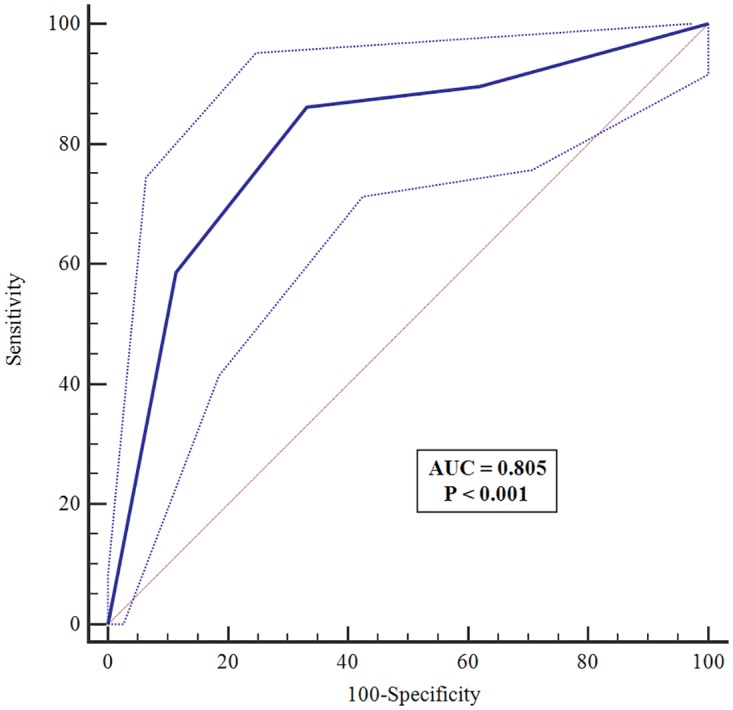
ROC curves of the Nepalese version of the MNA tool according to BMI. ROC, receiver operating characteristic; BMI, body mass index; AUC, area under ROC curve.

Spearman’s rank correlations between each dimensional item of the MNA and the total MNA score are given in S1 Table. Except for mobility, all items of the MNA were significantly correlated with the total score; many of the nutritional parameters showed high correlation (S1 Table). The total MNA score was significantly correlated with BMI (r = 0.58; p<0.001) (Fig 1). In the ROC curve analysis, based on BMI as a reference, diagnostic characteristics of the MNA- sensitivity, specificity and diagnostic accuracy—were satisfactory. A Youden-index cut-off value of 0.53 provided the highest sensitivity and specificity for the tool. At this cutoff, the MNA demonstrated sensitivity of 86% (95%CI: 75–94), but specificity was comparatively lower at 67% (95% CI: 60–74). The diagnostic accuracy of the MNA was 81% (95%CI: 75–85; *p* <0.001) (Fig 2).

### Nutritional assessment of participants

Given the validity of the MNA tool, the nutritional status of the participants in our study was analyzed. The mean (±SD) MNA score was 19.3 ± 4.2, which falls into the at-risk range. Only 11% (95%CI: 7.7–15.8) of the participants fell into the normal nutritional status range; 65% (95%CI: 58.6–70.7) were at risk of malnutrition, and 24% (95%CI: 19.0–29.8) were in the malnourished range of MNA scores (Table 2). Significant differences were found between nutritional status and several covariates: gender (*p* = 0.008), ethnicity (*p* = 0.02), education (*p* = 0.001), and marital status (*p* = 0.01). A significantly greater proportion of females, 29%, scored in the malnourished range than males, 18%. Forty percent of Dalit elderly were malnourished; only 7% of illiterates were of adequate nutritional status. Moreover, those who were married had better nutritional status than those who were separated or widowed. No significant differences in nutritional status were found by age (*p* = 0.40), religion (p = 0.22), smoking (*p* = 0.18), or alcohol consumption (*p* = 0.71) (Table 2).

**Table 2:** Nutritional status of participants and associated factors.

	Normal Nutritional Status n = 27, 11.2%	At Risk of Malnutrition n = 157, 64.9%	Malnourished n = 58, 24.0%	*p*–value^a^
**Total MNA score**^b^	25.43 ± 1.46	20.33 ± 2.07	13.68 ± 2.8	<0.001^a^
**BMI** ^b^	24.35 ± 3.22	22.05 ± 3.55	18.39 ± 3.39	<0.001^a^
**Age** ^b^	69.48 ± 3.78	69.39 ± 7.42	70.91 ± 8.59	0.4^a^
	**n (%)**	**n (%)**	**n (%)**	
**Gender**				
Male	19 (17.1)	72 (64.9)	20 (18.0)	0.008
Female	8 (6.1)	85 (64.9)	38 (29.0)	
**Ethnicity**				
Upper caste	19 (13.8)	84 (60.9)	35 (25.4)	0.02
Janjatis	5 (6.5)	60 (77.9)	12 (15.6)	
Dalit	3 (11.1)	13 (48.1)	11 (40.7)	
**Religion**				
Hindu	24 (13.3)	111 (61.3)	46 (25.4)	0.22
Buddhist	3 (5.2)	44 (75.9)	11 (19.0)	
Christian	0	2 (66.7)	1 (33.3)	
**Education**				
Literate	13 (24.5)	35 (66.0)	5 (9.4)	0.001
Informal	2 (14.3)	8 (57.1)	4 (28.6)	
Illiterate	12 (6.9)	114 (65.1)	49 (28.0)	
**Marital status**				
Married	25 (15.2)	108 (65.5)	32 (19.4)	0.01
Separated	-	8 (72.7)	3 (27.3)	
Widowed	2 (3.0)	41 (62.1)	23 (34.8)	
**Smoker**				
Never	11 (10.5)	71 (67.6)	23 (21.9)	0.18
Current	13 (14.0)	52 (55.9)	28 (30.1)	
Former	3 (7.0)	33 (76.7)	7 (16.3)	
**Drinker**				
Never	18 (10.7)	107 (63.7)	43 (25.6)	0.71
Infrequent	7 (11.3)	41 (66.1)	14 (22.6)	
Frequent	2 (16.7)	9 (75.0)	1 (8.3)	

^a^ ANOVA test for mean differences between nutritional status groups. All others are Chi-square tests.

^b^ Mean ± SD

Each of the 18 dimensions assessed by the MNA was also tested for significant association with the three categories of nutritional status. All but mobility (*p* = 0.85), independent living (*p* = 0.052), and protein intake (*p* = 0.77) were significantly associated with nutritional status category (S2 Table).

## Discussion

The MNA nutritional health screening tool has been validated in many settings [47–52]. However, although the MNA tool has been used in neighboring countries with socioeconomic and demographic characteristics closely resembling Nepal [25, 53–56], very few validation studies have been conducted in these regions. One validation study conducted in Kerala, India found the MNA-SF to be a valid tool for assessing nutritional status [57]. The current study validates the tool for the rural Nepal context.

The original developers of the MNA reported a higher sensitivity (96%) and specificity (98%) than the current study [20]. However, our findings of 86% sensitivity and 67% specificity are consistent with other studies that found high sensitivity but relatively low specificity of the tool, including studies conducted in Australia [58, 59], Brazil [49, 60], and Italy [47]. Differences in sensitivity and specificity can likely be attributed to variations in measurement accuracy of the MNA components for nutritional status, as well as regional differences in the use of comparison indicators. Nonetheless, existing literature supports that the MNA has high sensitivity and relatively lower specificity. In practice, this means that the tool is likely to have more “false positives” than “false negatives.” In the context of malnutrition, there is little harm in providing extra attention to an older person who has been falsely diagnosed as malnourished. Interventions are likely to include education, nutritional supplements, and additional health monitoring, which will not adversely impact “over diagnosed” patients. Therefore, this study supports the use of the MNA as an accurate and sensitive tool for nutritional assessment among the elderly in rural Nepal.

Using this tool, a high prevalence of malnutrition among the sampled elderly population in Okharpauwa was found. Nearly one-fourth of the participants were malnourished, with an additional 65% at risk of malnutrition. A technical brief published in 2012 reported similar findings among the elderly in the Pharping district of Nepal: 31% of the elderly were malnourished and 51% were at risk of malnutrition [61]. Taken together, these suggest a high burden of malnutrition among the Nepalese elderly (Table 3).

**Table 3:** The burden of malnutrition: Current and prior research results in South Asia among rural populations.

Location and Year of Study	Number of participants	Normal nutritional status	At risk of malnutrition	Malnourished
Okharpauwa, Nepal. 2016 (current)	242	11.2%	64.9%	24%
Pharping, Nepal. 2012 [61]	300	18%	51%	31%
Matlab, Bangladesh. 2006 [23]	457	12%	62%	26%
Assam, India. 2015 [25]	360	30%	55%	15%
Belagavi, India. 2016 [55]	190	33.7%	43.7%	22.6%

Other studies using the MNA conducted in different parts of South Asia suggest a similarly high prevalence of malnutrition among the elderly. Two studies conducted in India found prevalence of malnutrition at 15% and 23%. However, both had at least 30% of the population in the normal nutritional status range [25, 62], which is higher than the current study, with only 11% (Table 3). A 2006 nutritional assessment of rural elderly people in Bangladesh [23] revealed results most similar to those found in the current study, with 26% prevalence of malnutrition and 62% at risk of malnutrition (Table 3).

Consistent with our findings, a 2014 study conducted in urban Kolkata, India also showed that malnutrition was more prevalent among elderly females than males [62]. The gender disparity that is found in the prevalence of malnutrition among the elderly in this study, while not surprising, is an important finding that requires attention. Researchers have previously documented gender inequity in many aspects of Nepali society, including legal [63, 64], social [64], economic [65], educational [64, 66], and health [65]. Nepal ranks 98^th^ out of 187 countries on the United Nation’s Gender Inequity Index [67]. Yet, efforts to redress gender inequity are evident in Nepal’s participation in many international women’s rights treaties and forums [67], and the establishment of the Ministry of Women and Social Welfare in 1995 [68], among other efforts. Moreover, there are recent programs specifically targeted to nutrition for women. However, these primarily address women of child-bearing age, with a focus on reducing child mortality [68]; elderly women have not yet been included as a focus. In addition to diminished access to economic resources and education, Nepali women are adversely impacted by cultural practices as well. For example, traditionally, men and children are fed first, and given the more nutritious foods, often leaving women with little or no remaining nutritious food [69, 70]. Further research to determine the specific causes of this gender inequity in nutritional status of the elderly are warranted. Moreover, priority should be given promptly to elderly females for targeted nutrition interventions.

The association between ethnicity and malnutrition was also not surprising. The findings from this study are consistent with the 2012 study from the Pharping district of Nepal [61], which also identified ethnicity as a predictor of malnutrition among community-dwelling elderly. Researchers have shown a significant link between ethnicity and food security, with minority groups being most vulnerable [71–73]. In Nepal, the Dalit ethnic group, comprising 13% of the total Nepali population [4], have long been the most marginalized group due to historical, yet persistent, religious and cultural beliefs regarding social stratification of ethnic groups, previously referred to as castes [36, 74]. Consequently, most members of the Dalit ethnic group, historically considered the lowest caste, have low socioeconomic status, limited access to health, education, and employment, and high poverty [36]. Our study affirms a 2002 Situation Analysis Report for Dalits conducted in Nepal that found a high prevalence of malnutrition among the Dalit, especially high among Dalit women [36]. Culturally appropriate nutritional education programs and nutritional supplementation interventions are warranted to address this ethnic health disparity in Dalit elderly, who are documented here with inadequate nutrition.

In the current study, those with formal schooling had the best nutritional status. Education, often used as a proxy for socioeconomic status, is highly associated with many health outcomes [75]. It is likely that education is linked to the gender and ethnic disparities seen in the current study. Similarly, our finding of the protective effect of being married has been well documented [76, 77]. While the precise mechanisms by which marriage confers health benefits are unclear, studies show that married adults have better health and survival [76, 77]. Approaches to improve the nutritional status of the elderly should account for the literacy level of the targeted population; special attention to those who are not married may be warranted.

This study has several limitations. First, it was conducted among elderly people residing in a rural area of the country; thus, results may not be generalizable to those residing in urban areas. Households in rural areas are more likely to be food poor [78] and overall poverty rates are higher in rural areas of Nepal [79]; thus, the urban elderly populations may have better nutritional status. However, given that the vast majority of elderly currently reside in rural areas, the results here are important for immediate planning and action to address malnutrition. Additionally, the tool was only validated among community-dwelling elderly people, who represent the vast majority [5]. The extent to which the tool is valid among hospitalized and/or institutionalized elderly populations, as well as urban populations, should be explored in future studies, especially given that societal changes may make urban living and/or nursing home living more common among the elderly in Nepal.

Secondly, BMI was used as the standard marker for nutritional status for the purposes of tool validation. BMI has been noted for its limited applicability in nutritional assessment of obese individuals who are malnourished, and thus, may underestimate malnutrition [80]. However, as only four of 242 participants in the current study had a BMI ≥ 30, CDC’s classification for overweight and obese [81], it is unlikely that these findings are underestimated. Nonetheless, a future study validating the MNA against more rigorous standard, such as physician assessment accompanied by laboratory diagnosis of nutritional status, is desirable. Another limitation is that most dimensions of the MNA, specifically those related to dietary assessment, are measured by self-report, which may have had an impact on the current findings. Prior research has demonstrated that self-report of health behaviors tends to underestimate the true risk [82]. Given the social context for women in Nepal, it is quite likely that this underestimation would be stronger among women, possibly pointing to wider gender disparity as well as a greater overall prevalence of those at risk or malnourished. Lastly, as discussed previously, the specificity of the MNA was somewhat low, which means that the reported prevalence of malnutrition in this study may be overestimated.

Because the MNA has a short-form available, the MNA-SF, previously shown to be a valid and time-saving tool for the nutritional assessment of elderly [83, 84], it may be preferable to use this version in future nutritional assessments in Nepal. Therefore, separate analyses were conducted using only the six items found in the MNA-SF. Results were very similar between the full and short-form versions, and therefore the MNA-SF appears to be equally valid for assessing nutritional status of rural senior citizens in Nepal (S3 Table and S1 Fig).

This study suggests that the MNA is an accurate and sensitive tool for rapid nutritional screening of community-dwelling elderly populations in rural areas of Nepal. The high prevalence of malnutrition, now demonstrated by two studies in two different settings in Nepal, warrants conducting a comprehensive national assessment on the nutritional status of the burgeoning elderly population. At minimum, the incorporation of the elderly in existing ongoing nutritional surveys, including those targeting women of reproductive age [68], may be a cost-effective way to monitor elderly nutritional status nationwide. Furthermore, program interventions, including nutritional supplementation, education, and health monitoring for those with or at risk of malnutrition, should be developed and implemented by concerned authorities. Prompt attention should be focused on women and the Dalit ethnic group to reduce observed disparities in the prevalence of malnutrition in Nepal.

## Supporting information

S1 Table Item-total score correlations for the Nepalese version of the MNA. (DOCX)

S2 Table Nutritional status according to MNA dimensions. (DOCX)

S3 Table Item-total score correlations for the Nepalese version of the MNA-SF. (DOCX)

S1 Fig ROC curves of the Nepalese version of the MNA-SF tool according to BMI. ROC, receiver operating characteristic; BMI, body mass index; AUC, area under ROC curve.
(TIF)
