# Supplementary material for: Nutritional assessment of community-dwelling older adults in rural Nepal
Source: PLoS One. 2017 Feb 14;12(2):e0172052. doi: 10.1371/journal.pone.0172052 (PMC5308814; doi:10.1371/journal.pone.0172052)
Supplement: S1 Table — (DOCX) [file pone.0172052.s001.docx]

**S1 Table. Item-total score correlations for the Nepalese version of the MNA.**

| Item content | Spearman's rho, r | *p* -value |
| --- | --- | --- |
| Loss of appetite (past 3 months) | 0.40 | <0.001 |
| Weight loss (past 3 months) | 0.49 | <0.001 |
| Mobility | 0.06 | 0.36 |
| Psychological stress (past 3 months) | 0.48 | <0.001 |
| Neuropsychological problems | 0.49 | <0.001 |
| Body mass index (BMI) | 0.58 | <0.001 |
| Independent living | 0.21 | 0.001 |
| > 3 prescription drugs/day | 0.18 | 0.005 |
| Pressure sores or skin ulcers | 0.19 | 0.003 |
| Full meals/day | 0.35 | <0.001 |
| Protein intake | 0.22 | 0.001 |
| ≥ 2 servings of fruits/day | 0.29 | <0.001 |
| Daily fluid intake (cups) | 0.45 | <0.001 |
| Mode of feeding | 0.27 | <0.001 |
| Self-perceived nutritional status | 0.53 | <0.001 |
| Self-perceived health status | 0.37 | <0.001 |
| Mid-arm circumference | 0.29 | <0.001 |
| Calf circumference | 0.41 | <0.001 |

Abbreviations: MNA, mini nutritional assessment
