# Supplementary material for: Nutritional assessment of community-dwelling older adults in rural Nepal
Source: PLoS One. 2017 Feb 14;12(2):e0172052. doi: 10.1371/journal.pone.0172052 (PMC5308814; doi:10.1371/journal.pone.0172052)
Supplement: S2 Table — (DOCX) [file pone.0172052.s002.docx]

**S2 Table. Nutritional status according to MNA Dimensions.**

| MNA Dimensions | Characteristics | Normal nutritional  (n=27) | At risk of malnutrition  (n=157) | Malnourished  (n=58) | *p* -value |
| --- | --- | --- | --- | --- | --- |
|  |  | **n (%)** | **n (%)** | **n (%)** |  |
| Dietetic assessment | **Loss of appetite** | | | | |
|  | Severe decrease | - | 21 (46.7) | 24 (53.3) | <0.001 |
|  | Moderate decrease | 7 (8.9) | 52 (65.8) | 20 (25.3) |  |
|  | No decrease | 20 (16.9) | 84 (71.2) | 14 (11.9) |  |
|  | **Full meals/day** | | | | |
|  | 1 meal | - | 1 (50.0) | 1 (50.0) | <0.001 |
|  | 2 meals | 7 (5.6) | 72 (57.6) | 46 (36.8) |  |
|  | 3 meals | 20 (17.4) | 84 (73.0) | 11 (9.6) |  |
|  | **≥ 1 serving of dairy products/day (Yes)** | 16 (15.7) | 72 (70.6) | 14 (13.7) | 0.003 |
|  | **≥ 2 servings of fruits/day (Yes)** | 27 (13.8) | 132 (67.3) | 37 (18.9) | <0.001 |
|  | **Daily fluid intake (cups)** | | | | |
|  | < 3 | 3 (2.9) | 61 (58.1) | 41 (39.0) | <0.001 |
|  | 3-5 | 6 (8.0) | 56 (74.7) | 13 (17.3) |  |
|  | >5 | 18 (29.0) | 40 (64.5) | 4 (6.5) |  |
|  | **Mode of feeding** | | | | |
|  | Unable to self-feed | 1 (12.5) | 3 (37.5) | 4 (50.0) | 0.004 |
|  | Self-fed with some difficulty | - | 3 (30.0) | 7 (70.0) |  |
|  | Self-fed without any problem | 26 (11.6) | 151 (67.4) | 47 (21.0) |  |
| Anthropometric assessment | **BMI categories (Kg/m^2^)** |  |  |  |  |
|  | < 19 | - | 21 (38.2) | 34 (61.8) | <0.001 |
|  | 19 - < 21 | 3 (5.4) | 37 (66.1) | 16 (28.6) |  |
|  | 21 - < 23 | 5 (9.1) | 48 (87.3) | 2 (3.6) |  |
|  | ≥ 23 | 19 (25.0) | 51 (67.1) | 6 (7.9) |  |
|  | **Calf-circumference (cm)** |  |  |  |  |
|  | < 31 | 6 (3.8) | 102 (65.0) | 49 (31.2) | <0.001 |
|  | ≥ 31 | 21 (24.7) | 55 (64.7) | 9 (10.6) |  |
|  | **Mid-arm circumference (cm)** |  |  |  |  |
|  | < 21 | 1 (2.7) | 19 (51.4) | 17 (45.9) | <0.001 |
|  | 21 - 22 | 3 (4.8) | 41 (66.1) | 18 (29.0) |  |
|  | < 22 | 23 (16.1) | 97 (67.8) | 23 (16.1) |  |
|  | **Weight loss (past 3 months)** |  |  |  |  |
|  | Weight loss (>3 kg) | - | 19 (48.7) | 20 (51.3) | <0.001 |
|  | Weight loss (1 - 3 kg) | 3 (7.7) | 25 (64.1) | 11 (28.2) |  |
|  | Does not know | 4 (3.7) | 81 (74.3) | 24 (22.0) |  |
|  | No weight loss | 20 (36.4) | 32 (58.2) | 3 (5.5) |  |
| Global assessment | **Psychological** **stress (past 3 months)** | 1 (1.7) | 24 (41.4) | 33 (56.9) | <0.001 |
|  | **Neuropsychological problems** | | | | |
|  | Severe dementia | - | 5 (27.8) | 13 (72.2) | <0.001 |
|  | Mild dementia | 1 (1.1) | 61 (67.0) | 29 (31.9) |  |
|  | None | 26 (19.5) | 91 (68.4) | 16 (12.0) |  |
| Subjective assessment | **Self-perceived nutritional status** | | | | |
|  | Malnourished | - | 10 (23.8) | 32 (76.2) | <0.001 |
|  | Uncertain | 3 (5.3) | 44 (77.2) | 10 (17.5) |  |
|  | No nutritional problem | 24 (16.8) | 103 (72.0) | 16 (11.2) |  |
|  | **Self-perceived health status** | | | | |
|  | Not as good | 5 (6.0) | 41 (49.4) | 37(44.6) | <0.001 |
|  | Does not know | 5 (12.5) | 28 (70.0) | 7 (17.5) |  |
|  | As good | 12 (11.2) | 81 (75.7) | 14 (13.1) |  |
|  | Better | 5 (41.7) | 7 (58.3) | - |  |

Abbreviations: MNA, mini nutritional assessment; BMI, body mass index; kg, kilogram; m, meter; cm, centimeter
