# Supplementary material for: Nutritional assessment of community-dwelling older adults in rural Nepal
Source: PLoS One. 2017 Feb 14;12(2):e0172052. doi: 10.1371/journal.pone.0172052 (PMC5308814; doi:10.1371/journal.pone.0172052)
Supplement: S3 Table — (DOCX) [file pone.0172052.s003.docx]

**S3 Table. Item-total score correlations for the Nepalese version of the MNA-SF.**

| Item content | Spearman's rho, r | *p* -value |
| --- | --- | --- |
| Loss of appetite (past 3 months) | 0.51 | <0.001 |
| Weight loss (past 3 months) | 0.62 | <0.001 |
| Mobility | 0.10 | 0.105 |
| Psychological stress (past 3 months) | 0.48 | <0.001 |
| Neuropsychological problems | 0.60 | <0.001 |
| Body mass index (BMI) | 0.62 | <0.001 |

Abbreviations: MNA-SF, mini nutritional assessment short form
