# Supplementary figures and images for: Nutritional assessment of community-dwelling older adults in rural Nepal
Source: PLoS One. 2017 Feb 14;12(2):e0172052. doi: 10.1371/journal.pone.0172052 (PMC5308814; doi:10.1371/journal.pone.0172052)

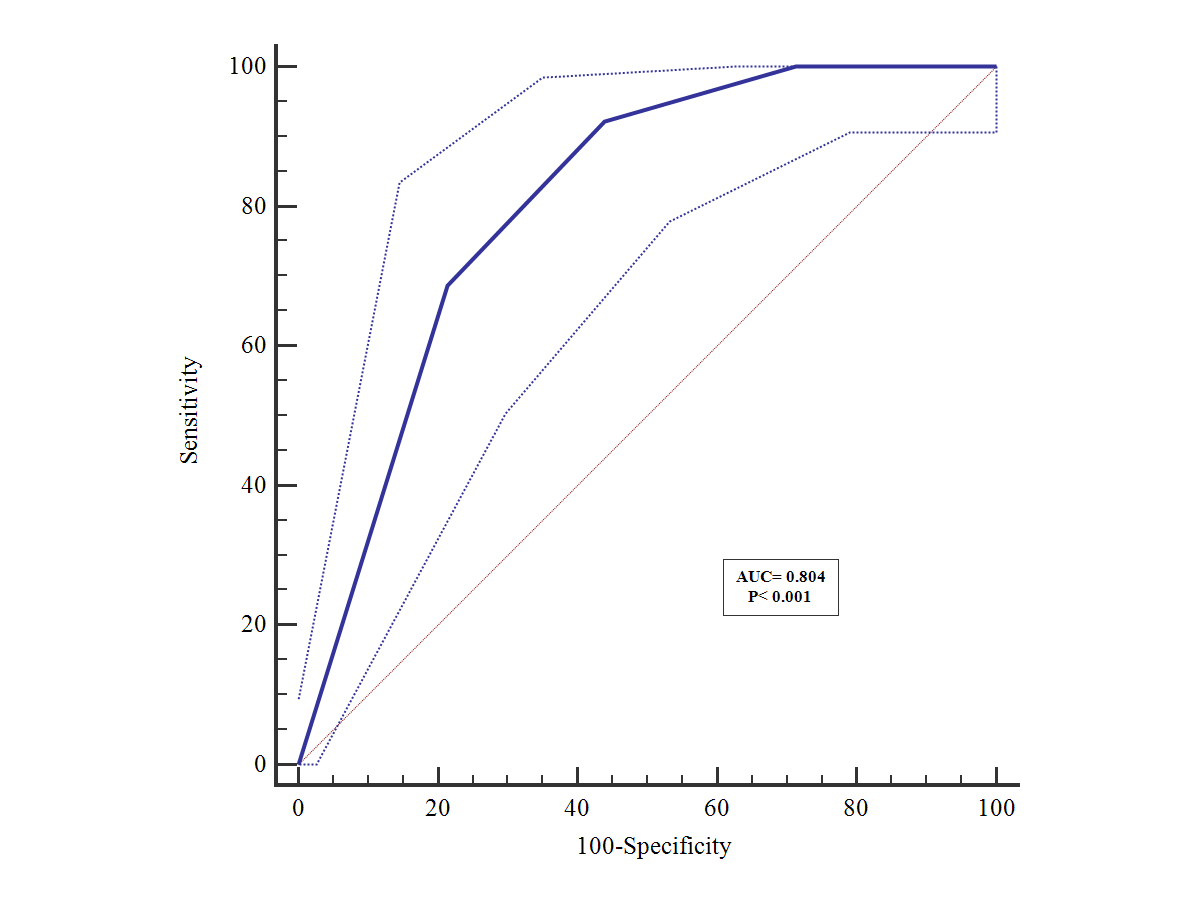

Supplement: S1 Fig — ROC, receiver operating characteristic; BMI, body mass index; AUC, area under ROC curve. (TIF) [file pone.0172052.s004.tif]
